# Characteristics of cerebral blood flow in an Eastern sample of multiple sclerosis patients: A potential quantitative imaging marker associated with disease severity

**DOI:** 10.3389/fimmu.2022.1025908

**Published:** 2022-10-17

**Authors:** Qinming Zhou, Tianxiao Zhang, Huanyu Meng, Dingding Shen, Yao Li, Lu He, Yining Gao, Yizongheng Zhang, Xinyun Huang, Hongping Meng, Biao Li, Min Zhang, Sheng Chen

**Affiliations:** ^1^ Department of Neurology and Institute of Neurology, Ruijin Hospital, Shanghai Jiao Tong University School of Medicine, Shanghai, China; ^2^ School of Biomedical Engineering, Shanghai Jiao Tong University, Shanghai, China; ^3^ Department of Nuclear Medicine, Ruijin Hospital, Shanghai Jiao Tong University School of Medicine, Shanghai, China; ^4^ Co-innovation Center of Neuroregeneration, Nantong University, Nantong, China; ^5^ Department of Neurology, Xinrui Hospital, Wuxi, China

**Keywords:** cerebral blood flow, multiple sclerosis, quantitative imaging marker, disease severity, EDSS, eastern population, neuroimaging

## Abstract

Multiple sclerosis (MS) is a chronic inflammatory disease of the central nervous system that is rare in China. At present, there are no widespread quantitative imaging markers associated with disease severity in MS. Despite several previous studies reporting cerebral blood flow (CBF) changes in MS, no consensus has been reached. In this study, we enrolled 30 Eastern MS patients to investigate CBF changes in different brain regions using the arterial spin labeling technique and their relationship with disease severity. The average CBF in MS patients were higher than those in health controls in various brain regions except cerebellum. The results indicated that MS patients with strongly increased CBF showed worse disease severity, including higher Expanded Disability Status Scale (EDSS) scores and serum neurofilament light chain (sNfL) values than those with mildly increased CBF in the parietal lobes, temporal lobes, basal ganglia, and damaged white matter (DWM). From another perspective, MS patients with worse disease severity (higher EDSS score and sNfL values, longer disease duration) showed increased CBF in parietal lobes, temporal lobes, basal ganglia, normal-appearing white matter (NAWM), and DWM. Correlation analysis showed that there was a strong association among CBF, EDSS score and sNfL. MS patients with strongly increased CBF in various brain regions had more ratio in relapsing phase than patients with mildly increased CBF. And relapsing patients showed significantly higher CBF in some regions (temporal lobes, left basal ganglia, right NAWM) compared to remitting patients. In addition, MS patients with cognitive impairment had higher CBF than those without cognitive impairment in the right parietal lobe and NAWM. However, there were no significant differences in CBF between MS patients with and without other neurologic dysfunctions (e.g., motor impairment, visual disturbance, sensory dysfunction). These findings expand our understanding of CBF in MS and imply that CBF could be a potential quantitative imaging marker associated with disease severity.

## Introduction

Multiples sclerosis (MS) is a chronic inflammatory disease of the central nervous system characterized by progressive neurologic degeneration, eventually leading to irreversible disability and impaired cognition in around 60% of patients (1). It is known that MS occurs mostly in high latitude regions, such as northern Europe. However, MS is still a rare disease in the Chinese population. The reported age- and sex-adjusted incidence of MS from population surveys in China is 0.235 per 100,000 person-years (2). The mean age at onset in Chinese MS patients is around 30 years, with few cases younger than 20 years (1, 2). Because of the young age of onset and inability to work, the disease burden caused by MS is considered heavy.

Although studies have proposed various imaging measures and laboratory markers as biomarkers for diagnosis and clinical assessment of MS, these biomarkers are not available for widespread clinical use due to their poor correlation with clinical disability (3). Useful biomarkers will likely emerge that will enable us to assess clinical severity and predict and monitor disease progression. At present, magnetic resonance imaging (MRI) is the most important tool for diagnosis and prognosis evaluation of MS. White matter (WM) lesions in MS patients can be visualized by T2W MRI, and acute inflammation can be detected by gadolinium contrast enhancement due to the breach of the blood-brain barrier. Besides damaged white matter (DWM), the widespread and subtle changes occur in normal-appearing white matter (NAWM) and grey matter (GM), which may be accompanied by alterations in vascular function (4). Although growing evidence suggests that vascular aspects play an important role in the disease, brain perfusion changes in MS patients have received little attention (5). Brain perfusion is primarily observed by the measurement of cerebral blood flow (CBF). With the development of imaging technology, researchers can use non-invasive methods to safely and accurately assess intracranial blood flow difference in MS patients. Dynamic susceptibility contrast-enhanced MRI, arterial spin labeling (ASL) technique, computed tomography, and radionuclide imaging can be used to provide cerebral perfusion parameters (6). Despite several studies about brain perfusion changes in MS patients, no consensus has been reached. Some studies reported hypoperfusion in DWM, NAWM, and GM in MS patients (7, 8), while others reported increased CBF in multiple brain regions (9, 10).

Although there is agreement that perfusion changes represent an important component of MS process, the relationship between CBF changes and clinical parameters in MS, such as Expanded Disability Status Scale (EDSS), annualized relapse rate (ARR), motor impairment, and cognitive impairment, is still not clear. Moreover, the CBF distribution in different brain regions in MS patients is varied. It remains unclear if CBF in different brain regions changes with clinical parameters. Thus, correlation analysis between CBF in different brain regions and clinical parameters can provide clues for the evaluation of clinical severity and therapeutic effects. In addition, research on Eastern populations is limited (11). Zhang et al. found that MS patients had reduced CBF in the occipital cortex and increased CBF in the right putamen (11). The potential value of CBF changes in MS remains controversial and poorly understood.

To shed light on these questions, this study evaluated CBF characteristics in a sample of Eastern patients with MS to comprehensively study its association with disease severity and multiple clinical parameters for the first time.

## Methods

### Patients

A total of 30 Eastern MS patients and eight healthy controls (HCs) participated in this study. The inclusion criteria were as follows: 1) being a healthy individual or having a MS diagnosis according to the revised 2017 McDonald criteria and a relapsing-remitting course; 2) between 18–60 years old. The exclusion criteria were: 1) cardiovascular and/or metabolic diseases; 2) psychiatric disorders and/or neurologic disease other than MS; 3) body mass index (weight/height^2^) higher than 30; or 4) pregnancy. Disability in MS patients was quantified with EDSS administered by an experienced neurologist within two weeks of MRI scan. Cognitive status was assessed using Montreal Cognitive Assessment (MoCA) test and Mini-Mental State Examination (MMSE). Cognitive impairment was defined as both MoCA score < 26 and MMSE score < 25. Motor function was assessed with muscle strength testing based on Medical Research Council (MRC) grades. Patients were classified as motor impaired if they had muscle strength weakness. Follow-up was conducted 18 months after imaging examinations to observe relapse. The study was approved by the Ethics Committee of Ruijin Hospital, and it was performed in accordance with the principles of the Helsinki Declaration. Written informed consent was obtained from all participants.

### MRI protocol and data processing

MRI acquisition was performed using a Biograph mMR system (Siemens, Erlangen, Germany). Single inverse time 2D pulsed arterial spin labeling (pASL) data were acquired with the following parameters: bolus duration = 1675 ms, inverse time (TI) = 1800 ms, repetition time (TR) = 2500 ms, echo time (TE) = 11 ms, flip angle (FA) = 90°, field of view = 192 × 192 mm^2^, voxel size = 3 × 3 × 5 mm^3^, slices = 16, measurements = 150, and acquisition time = 6:27 min.

ASL data preprocessing was performed to eliminate systematic and subject-related bias before CBF quantification. First, motion correction was applied on labeled and control images using MCFLIRT tool found in the FMRIB’s Software Library (FSL) (12). To reduce intensity inhomogeneity in the coil sensitivity, we implemented the field correction algorithm termed N4ITK in Advanced Normalization Tools (13).

DWM tissue was segmented on MNI space T2-weighted image using a lesion prediction algorithm in the Lesion Segmentation Tool (LST) toolbox (14). For NAWM tissue segmentation, we first performed bias correction algorithm on MNI space T1 images using N4ITK before the WM tissue segmentation (13). A unified segmentation model in Statistical Parametric Mapping (SPM12) software was performed on unbiased 3D T1 images for tissue segmentation (15). WM tissue probabilistic maps could be obtained. Regions where the probabilistic map is greater than 0.95 and is not DWM are defined as NAWM. We checked each segmentation results after the segmentation process.

CBF quantification included the following steps: 1) To eliminated static tissue signal, seventy-five difference images were obtained by subtracting the labeled images from the control image. 2) Perfusion-weighted images were generated by averaging the difference images. 3) Relative CBF images were calculated by general kinetic modelling using the BASIL FSL toolbox (16). 4) Absolute CBF was calibrated by surrogate M_0_ images calculated from the averaged control images (17).

Quantitative CBF images were registered to MNI space using FMRIB’s Linear Image Registration Tool and FMRIB’s Nonlinear Image Registration Tool for statistical analysis (18). Firstly, ASL space T1 image was obtained by linear registration of 3D-T1 image into surrogate M0 images calculated from the averaged control ASL images to compensate deviation due to head motion. Secondly, ASL space T1 image was used to generate the deformation field from ASL space to MNI space. Finally, we applied the derived deformation field to ASL space quantitative CBF images, thus obtaining MNI space quantitative CBF images. We double checked the registration results of each step using ITK-SNAP (19). All the MNI space quantitative CBF image were well aligned with MNI template.

### Statistical analysis

Statistical analysis was performed using SPSS 19.0 (IBM, New York, USA). A *p*-value of < 0.05 was considered statically significant. Categorical data were compared using the χ^2^ test. Group comparisons were conducted *via* the paired t test or analysis of variance (ANOVA). In cases where the variance between groups was equal (*p* > 0.05) or unequal, the LSD test or Dunnett’s *t*-test was performed, respectively. Correlations were assessed using Spearman correlation. The data were presented as the mean ± standard deviation (SD).

## Results

### Demographic and diseases characteristics

A total of 30 MS patients and 8 HCs were enrolled in the study. There were no significant differences in sex or age between MS patients and HCs. The average age was 40.07 ± 11.20 years in MS patients and 34.00 ± 6.55 years in HCs. The MS patients group included 16 males and 14 females, while the HC group included 4 males and 4 females. The longest disease duration was 396 months and the shortest was 23 months. The EDSS score ranged from 1 to 7. The clinical data for MS patients and HCs are shown in **Table 1**.

**Table 1:** Summary of MS patients and HCs.

	MS patients	HC
Number	30	8
Age, year	40.07 ± 11.20	34.00 ± 6.55
Gender (male/female)	16/14	4/4
EDSS	2.97 ± 1.80	/
EDSS ≤ 3 (n)	19	/
EDSS > 3 (n)	11	/
Disease duration (month)	85.30 ± 90.29	/
Duration ≤ 36 (n)	14	/
Duration > 36 (n)	16	/
sNfL (pg/ml)	20.25 ± 7.10	/
Relapsing phase (n)	12	/
With new relapsing occurrences	9	
ARR	0.54 ± 0.27	/
Cognitive impairment (n)	8	/
Motor impairment (n)	11	/
Sensory disturbance (n)	7	/
Visual impairment (n)	6	/

### Regional distribution of CBF in MS patients

**Figure 1:**
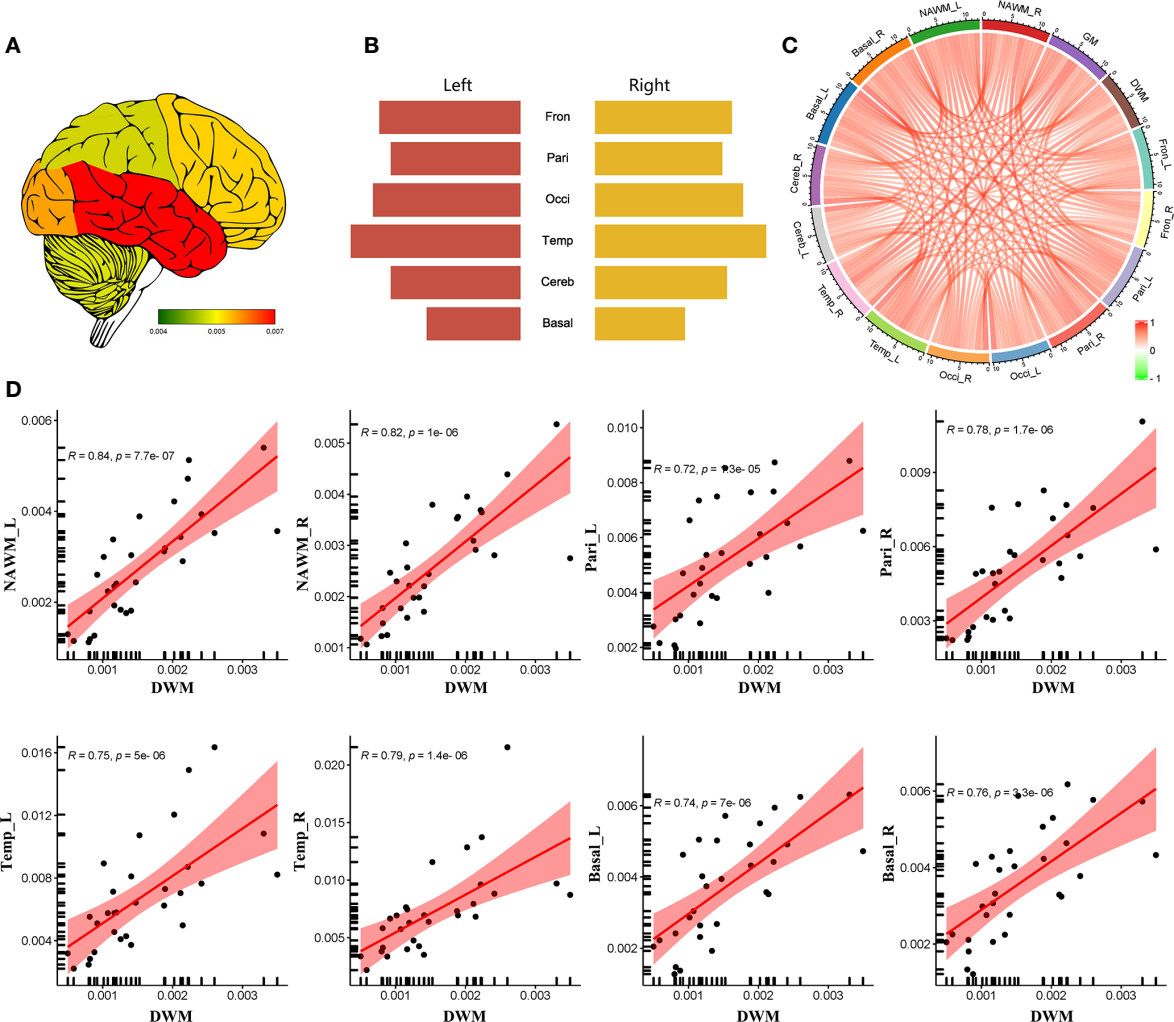
The regional distribution of CBF in MS patients. **(A)** CSF heatmap of different brain regions. **(B)** Comparison of CBF in different brain regions. **(C)** Correlation analysis among different regions. **(D)** Significantly positive correlation of CBF between DWM and some brain region (NAWM, parietal lobes, temporal lobes and basal ganglia). (Fron_L, left frontal lobe; Fron_R, right frontal lobe; Pari_L, left parietal lobe; Pari_R, right parietal lobe; Occi_L, left occipital lobe; Occi_R, right occipital lobe; Temp_L, left temporal lobe; Temp_R, right temporal lobe; Cereb_L, left cerebellum; Cereb_R, right cerebellum; Basal_L, left basal ganglia; Basal_R, right basal ganglia; NAWM_L, left normal-appearing white matter; NAWM_R, right normal-appearing white matter; DWM, damaged white matter).

The lowest CBF in the whole brain was observed in DWM (0.0018 ± 0.0012). CBF in NAWM was significantly higher than that in DWM (*p* = 0.0024). Among different regions of GM, the highest CBF was in the right temporal lobe (0.0073 ± 0.0039), and the lowest CBF was in the right basal ganglia (0.0036 ± 0.0014) (**Figures 1A, B**).

Correlation analysis indicated that CBF values in DWM, NAWM, and GM were positively correlated with each other (**Figure 1C**). In addition, CBF in some GM regions (e.g., bilateral parietal lobes, temporal lobes, and basal ganglia) and NAWM showed a significantly positive correlation with CBF in DWM (**Figure 1D**). These findings indicate that NAWM and GM in MS patients show insidious changes.

### The comprehensive investigation of CBF changes in MS patients

The patients were divided into different groups respectively (high vs. low EDSS; high vs. low serum neurofilament light chain (sNfL); long vs. short disease duration; high ARR vs. low ARR) based on the median value. We compared CBF among patients with different clinical parameters (high vs. low EDSS; high vs. low sNfL; long vs. short disease duration; in relapsing vs. remitting phase; high ARR vs. low ARR; with vs. without cognitive impairment, motor impairment, visual disturbance, or sensory dysfunction).

**Figure 2:**
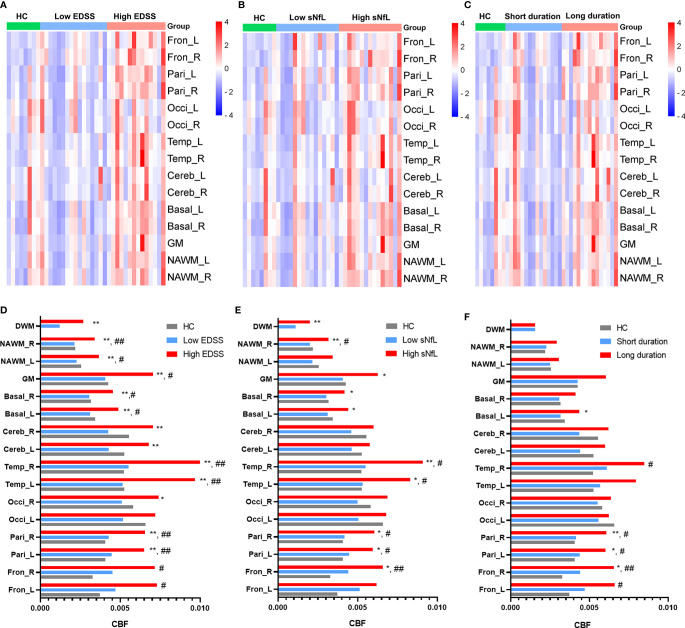
Relationship between CBF and clinical parameters. Comparisons of CBF differences in different regions among: **(A)** HCs, MS patients with low EDSS scores, and MS patients with high EDSS scores; **(B)** HCs, MS patients with low sNfL, and MS patients with high sNfL; and **(C)** HCs, MS patients with short disease duration, and MS patients with long disease duration. **(D–F)** Comparison of CBF of different regions according to clinical parameters (EDSS, sNfL, and disease duration). *p < 0.05 and **p < 0.01 vs. low EDSS group/short duration group/low sNfL group; #*p* < 0.05 and ##*p* < 0.01 vs. HCs.

CBF was higher in the DWM, NAWM, basal ganglia, parietal lobes, and frontal lobes of patients with high EDSS scores (≥ 3, n = 14) compared to those with low EDSS scores (< 3, n = 16) and HCs (**Figures 2A, D**). However, there were no significant differences in CBF between patients with low EDSS scores and HCs. And MS patients with high sNfL had increased CBF in DWM, bilateral NAWM, GM, bilateral basal ganglia, bilateral temporal lobes, bilateral parietal lobes, and right frontal lobe compared to those with low sNfL (**Figures 2B, E**).

MS patients with a long disease duration (> 36 months, n = 15) had higher CBF in bilateral parietal lobes (left: *p* = 0.19, right: *p* = 0.009), right basal ganglia (*p* = 0.035), and right frontal lobes (*p* = 0.020) than those with a short disease duration (≤ 36 months, n = 15) and HCs (**Figures 2C, F**). However, MS patients with a short disease duration did not show obvious CBF changes compared with HCs.

**Figure 3:**
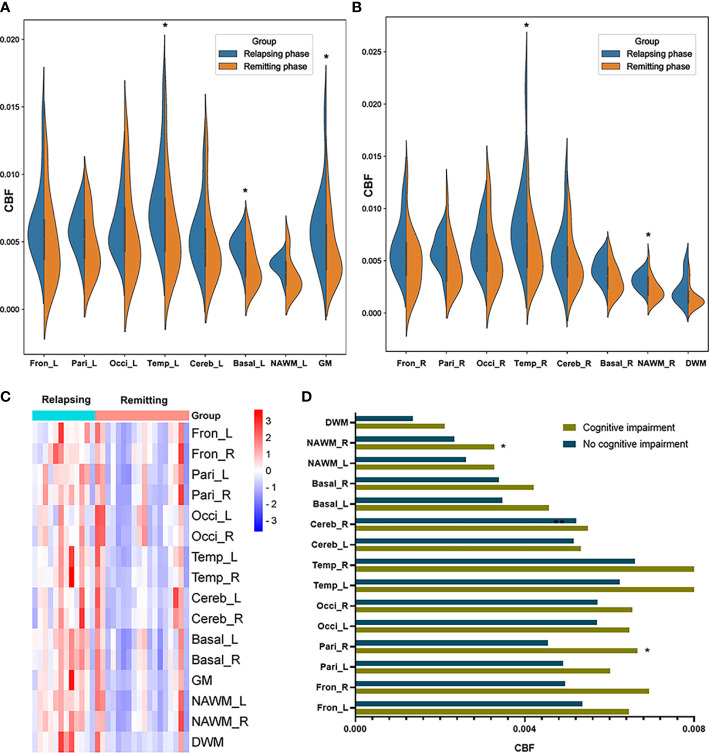
CBF differences between MS patients in the relapsing phase and remitting phase, and between MS patients with and without cognitive impairment. **(A, B)** Comparison of CBF between MS patients in the relapsing phase and remitting phase; **(C)** CBF cluster heatmap of different regions in different phases; and **(D)** MS patients with cognitive impairment showed increased CBF in the right parietal lobe and right NAWM compared to those without. *p < 0.05 vs. patients in remitting phase/without cognitive impairment.

Except for DWM, CBF values were affected in patients in the relapsing phase. Specifically, the bilateral temporal lobes (left: *p* = 0.033, right: *p* = 0011), right NAWM (*p* = 0.029), and left basal ganglia (*p* = 0.018) CBF values were significantly increased in relapsing patients compared to remitting patients (**Figures 3A–C**).

We then tested whether MS patients with different neurologic dysfunctions showed abnormal CBF in multiple brain regions. Relative to MS patients without cognitive disorder (n = 22), MS patients with cognitive disorder (n = 8) had increased CBF in right parietal lobes (*p* = 0.016) and right NAWM (*p* = 0.029) (**Figure 3D**). We compared CBF in MS patients with/without some other neurologic dysfunctions (motor impairment, visual disturbance, sensory dysfunction). However, no significant differences were found in these comparisons. We equally divided MS patients into two groups (high ARR group and low ARR group) according to ARR values. There were no significant differences between two groups in various brain regions.

### Association of CBF data with MS clinical severity

**Figure 4:**
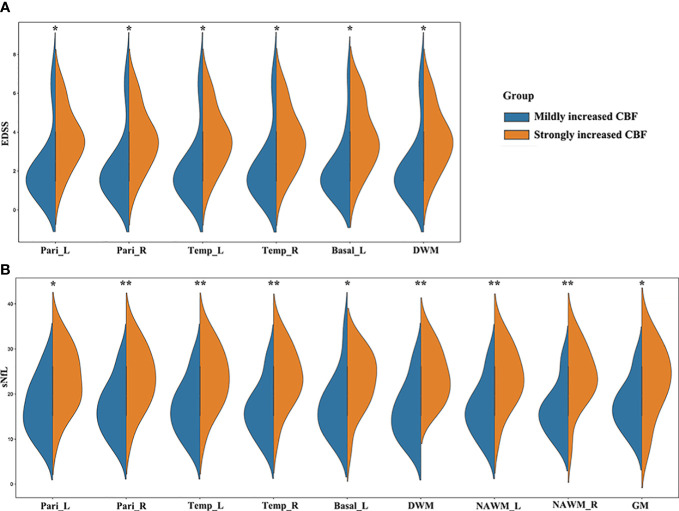
MS patients with different CBF values showed different clinical severity. **(A)** EDSS scores in MS patients with strongly increased CBF were significantly higher than those in patients with mildly increased CBF in the bilateral parietal lobes, bilateral temporal lobes, left basal ganglia, and DWM; **(B)** MS patients with strongly increased CBF had significantly higher sNfL than patients with mildly increased CBF in the bilateral parietal lobes, bilateral temporal lobes, left basal ganglia, DWM, bilateral NAWM, and global GM. *p < 0.05 and **p < 0.01 vs. mildly increased CBF group.

**Figure 5:**
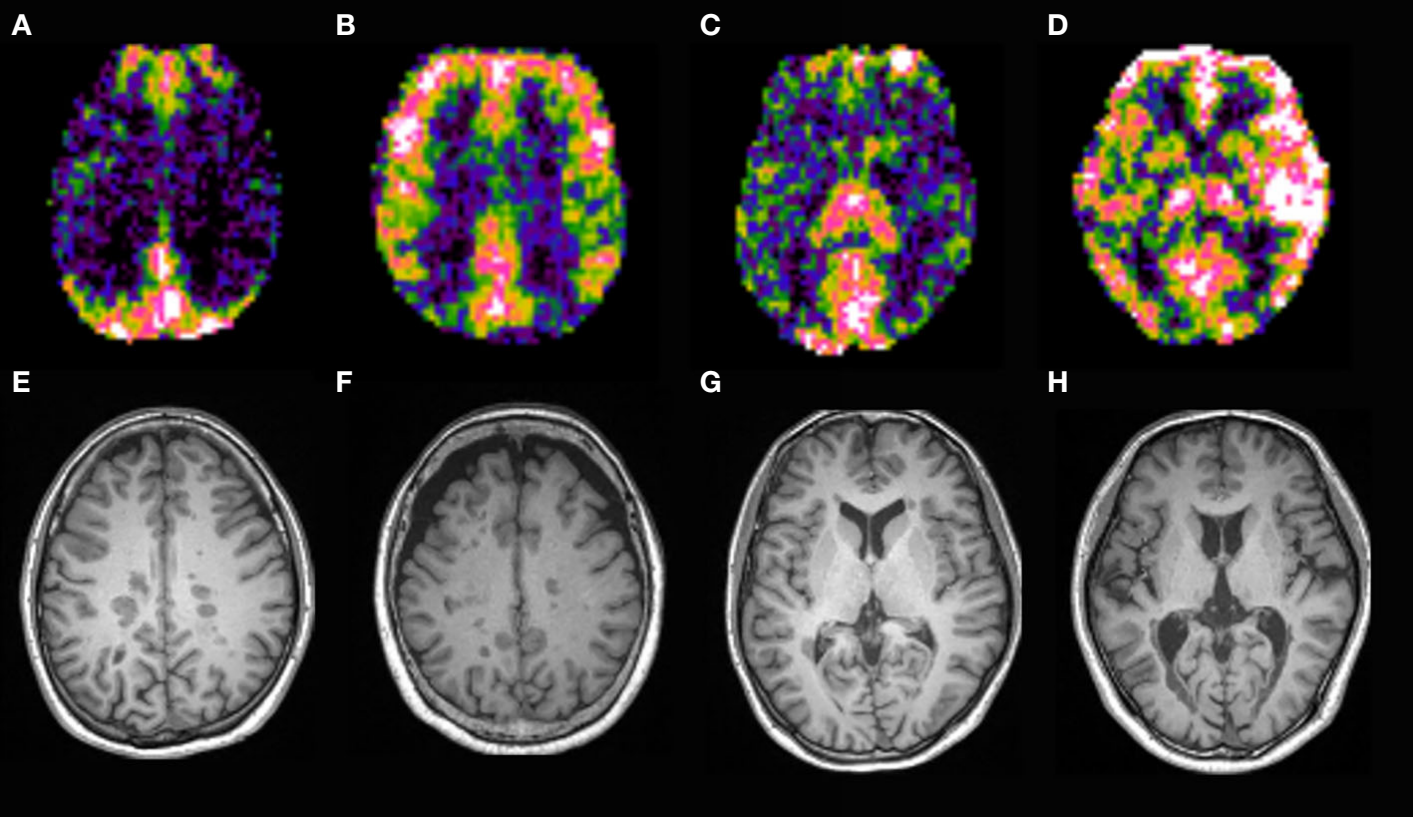
CBF map of MS patients. Patients with strongly increased CBF in the parietal lobes **(B, F)** had higher EDSS and sNfL than those with mildly increased CBF **(A, E)**. Patients with strongly increased CBF in the temporal lobes and basal ganglia **(D, H)** had higher EDSS and sNfL than those with mildly increased CBF **(C, G)**.

Comparing with HCs, MS patients had significantly increased CBF in right frontal lobe (p = 0.039). Although there were no significant differences, MS patients had slightly higher CBF than HCs in the other brain regions except cerebellum. The 30 MS patients were equally divided into strongly increased CBF (CBF > median, n = 15) and mildly increased CBF (CBF < median, n = 15) groups based on the median CBF in different regions. EDSS scores in MS patients with strongly increased CBF were significantly higher than those in patients with mildly increased CBF in some regions (e.g., bilateral parietal lobes, bilateral temporal lobes, left basal ganglia, and DWM) (**Figures 4A**, **5**). In MS patients, elevation in sNfL is linked to worse neurologic function, greater clinical disability, and lower brain volumes (20, 21). Our results showed that MS patients with strongly increased CBF had significantly higher sNfL than patients with mildly increased CBF in some regions, namely the bilateral parietal lobes, bilateral temporal lobes, left basal ganglia, DWM, NAWM, and global GM (**Figures 4B**, **5**).

**Figure 6:**
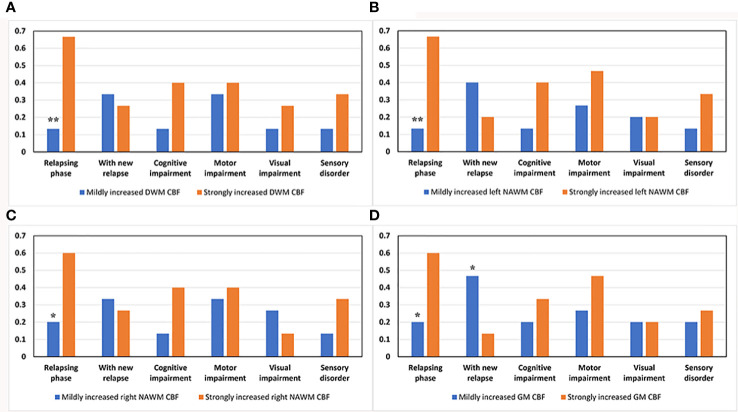
Comparison of various clinical parameters between the mildly and strongly increased CBF groups in DWM, NAWM, and GM. The strongly increased CBF group had a higher ratio of patients in the relapsing phase than the mildly increased CBF group in **(A)** DWM (*p* = 0.003), **(B)** left NAWM (*p* = 0.003), **(C)** right NAWM (*p* = 0.025), and **(D)** global GM (*p* = 0.025). **(D)** The low GM CBF group had a higher ratio of patients experiencing relapse after the neuroimaging examination than the high GM CBF group (*p* = 0.046). *p < 0.05 and **p < 0.01 vs. strongly increased group.

Next, we used the χ^2^ test to compare the ratio of MS patients in the relapsing phase, with cognitive impairment, motor impairment, visual disorder, sensory disturbance, and with new relapsing occurrences in 18-month follow-up between the strongly and mildly increased CBF groups for global GM, DWM, and NAWM. Of 30 MS patients, 12 were in the relapsing phase when they underwent the neuroimaging examination and 18 were in the remitting phase. The strongly increased CBF group had a significantly higher ratio of relapsing MS patients than the mildly increased CBF group in global GM, DWM, and NAWM (**Figure 6**). At 18-months follow-up after the neuroimaging examination, nine MS patients suffered from relapse. The low GM CBF group had more patients experiencing relapse than the high GM CBF group (**Figure 6D**). However, there were no significant differences in cognitive impairment, motor impairment, visual disorder, or sensory disturbance (**Figure 6**). Furthermore, the strongly and mildly increased CBF groups showed no significant differences in ARR for the brain regions.

**Figure 7:**
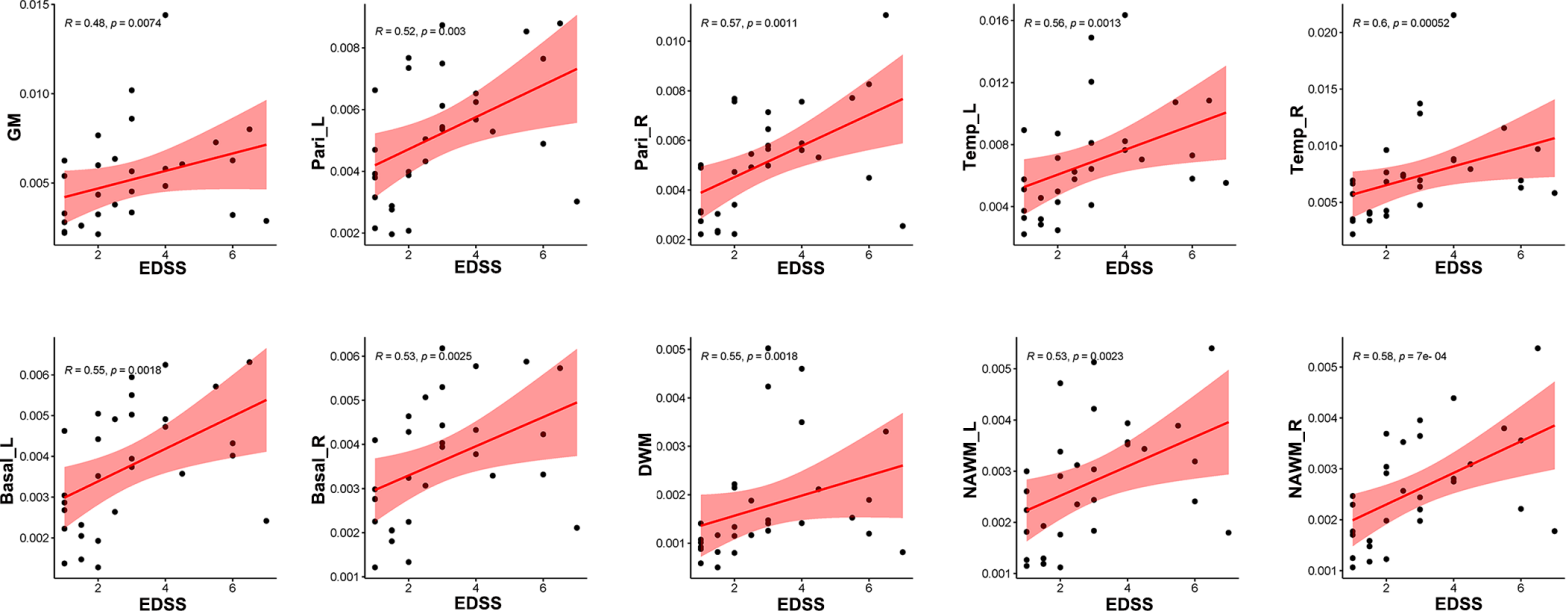
Correlation between EDSS scores and CBF in different regions. CBF in global GM, bilateral parietal lobes, bilateral temporal lobes, bilateral basal ganglia, DWM and NAWM were positively related with EDSS sores.

**Figure 8:**
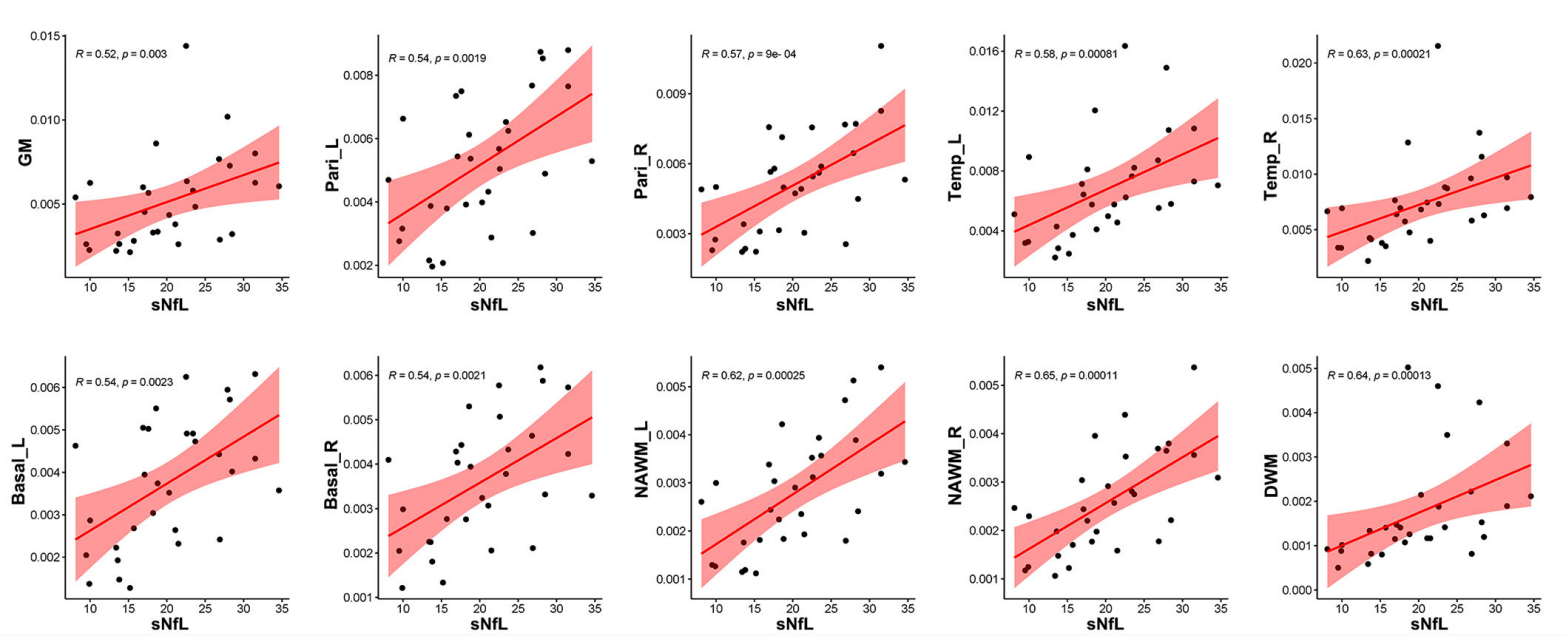
Correlation between sNfL and CBF in different regions. CBF values in global GM (*R* = 0.52, *p* = 0.0030), bilateral parietal lobes (left: *R* = 0.54, *p* = 0.0019, right: *R* = 0.57, *p* = 0.0009), bilateral temporal lobes (left: *R* = 0.58, *p* = 0.00081, right: *R* = 0.63, *p* = 0.00021), bilateral basal ganglia (left: *R* = 0.54, *p* = 0.0023, right: *R* = 0.54, *p* = 0.0021), NAWM (left: *R* = 0.62, *p* = 0.00025, right: *R* = 0.65, *p* = 0.00011), and DWM (*R* = 0.64, *p* = 0.00013) were positively related to sNfL.

Correlation analysis revealed that CBF in GM (*R* = 0.48, *p* = 0.0074), bilateral parietal lobes (left: *R* = 0.52, *p* = 0.003, right: *R* = 0.57, *p* = 0.0011), bilateral temporal lobes (left: *R* = 0.56, *p* = 0.0013, right: *R* = 0.60, *p* = 0.00052), bilateral basal ganglia (left: *R* = 0.55, *p* = 0.0018, right: *R* = 0.53, *p* = 0.0025), DWM (*R* = 0.55, *p* = 0.0018), and NAWM (left: *R* = 0.53, *p* = 0.0023, right: *R* = 0.58, *p* = 0.0007) were positively correlated with EDSS score (**Figure 7**), while CBF in GM (*R* = 0.52, *p* = 0.003), bilateral parietal lobes (left: *R* = 0.54, *p* = 0.0019, right: *R* = 0.57, *p* = 0.0009), bilateral temporal lobes (left: *R* = 0.58, *p* = 0.00081, right: *R* = 0.63, *p* = 0.00021), bilateral basal ganglia (left: *R* = 0.54, *p* = 0.0023, right: *R* = 0.54, *p* = 0.0021), bilateral NAWM (left: *R* = 0.62, *p* = 0.00025, right: *R* = 0.65, *p* = 0.00011) and DWM (*R* = 0.64, *p* = 0.00013) were positively correlated with sNfL (**Figure 8**).

## Discussion

### Limitations of conventional MRI in MS

MS is a chronic disabling disease of the central nervous system characterized by the presence of focal white matter lesions, inflammation, demyelination, and axon loss. The most important method for MS diagnosis is MRI, which can interrogate the entire central nervous system. Conventional MRI (T1WI and T2WI) can help assess lesion load and identify acute lesions, thus providing valuable information for diagnosis, assessment of disease severity, and treatment effect. However, it cannot provide quantitative analysis of brain function in MS patients and does not correlate strongly with clinical status (22, 23). Except the pathologic changes in DWM, growing evidence suggests a more widespread and subtle form of disease activity also occurs in NAWM and GM (24, 25), which may have an association with clinical manifestation. Conventional MRI is not sensitive to the pathology affecting CNS tissue outside DWM (i.e., in NAWM and GM).

### Progress in quantitative imaging for MS

Due to the limitations of conventional MRI, some quantitative imaging techniques have been developed, such as positron emission tomography (PET), diffusion tensor imaging (DTI), perfusion MRI, relaxometry, myelin imaging, and magnetization transfer, that could provide better quantification of the extent, type, spatial distribution, and evolution of CNS tissue damage in MS. These methods could contribute to better assessment of disease severity, therapy response, and stratification of disease burden. Furthermore, some quantitative imaging techniques are more sensitive to subtle alterations within or outside lesions compared with conventional MRI. Our previous study demonstrated that dynamic ^18^F-florberapir PET is a promising tool for quantitatively monitoring myelin loss and recovery and correlates closely with changes in EDSS (14, 26). DTI can be used to assess CNS tissue integrity and provide information on microstructure changes of DWM and NAWM in MS patients (14). Previous studies have shown that brain perfusion changes play an important role in MS pathophysiology and precede the initial blood-brain barrier breakdown and T2-weighted lesion appearance (23, 27). Theoretically, brain perfusion MRI has the ability to quantitatively assess disease status in MS. However, the relationship between brain perfusion and clinical parameters is still controversial (7, 8, 10, 28, 29). As these quantitative imaging techniques have not reached clinical maturity, they are not widely used in clinical practice.

### Characteristics of CBF in an Eastern sample of MS patients and their relationship with clinical parameters

In this study, we first comprehensively investigated CBF changes in Eastern MS patients using the ASL technique. We observed CBF changes in different brain regions, including DWM, NAWM, frontal lobes, parietal lobes, temporal lobes, occipital lobes, basal ganglia, and cerebellum. The relationship between CBF changes in various brain regions and multiple clinical parameters (EDSS, sNfL, relapsing/remitting phase, duration, cognitive disorder, ARR, motor impairment, sensory disorder, visual disturbance) was then explored. MS patients with strongly increased CBF in parietal lobes, temporal lobes, and basal ganglia had worse clinical disability (higher EDSS scores and higher sNfL) than those with mildly increased CBF. Increased CBF could represent a physiologic response to an increased demand that was secondary to increased inflammatory, and glial cell activity (6, 30). In an inflammatory environment, the inducible form of NOS (iNOS) is upregulated, producing more nitric oxide (NO) and nitrogen reactive species. NO has the effect of vasodilation and can alter BBB (31).

In addition, the group with strongly increased CBF in DWM, NAWM, and GM had more patients in the relapsing phase compare to the group with mildly increased CBF in these regions, and the ratio of patients experiencing relapse at 18-month follow-up was higher among the group with low GM CBF relative to high GM CBF. At the same time, CBF in patients with serious disability (high EDSS scores and high sNfL) was higher than that in those with mild disability (low EDSS scores and low sNfL) in multiple regions (e.g., DWM, NAWM, parietal lobes, temporal lobes, and basal ganglia). There was a strong correlation between EDSS scores, sNfL values, and CBF changes in these regions.

Furthermore, we found that patients in the relapsing phase, with a long disease duration, or with cognitive impairment had higher CBF in certain brain regions (e.g., parietal lobes, temporal lobes, basal ganglia and NAWM) than those in the remitting phase, with a short disease duration, or without cognitive impairment. In contrast, no significant differences were found in comparisons of other clinical phenotypes (ARR, motor impairment, sensory disorder, visual disturbance). These findings demonstrate that CBF is closely associated with MS clinical severity.

### The potential of CBF as a quantitative imaging marker of disease severity

In MS, the identification of reliable imaging biomarkers will facilitate early diagnosis and monitoring of disease severity (14). Although some imaging biomarkers have been proposed, various challenges limit their widespread adoption in clinical practice (32, 33). For example, brain atrophy is strongly related to disease progression. However, detection of brain atrophy is dependent on complex measuring methods and long-term observation, and it is not suitable for the prediction of early progression (34). Although conventional MRI remains the main imaging technique in MS, quantitative imaging techniques show emerging applications. In the present study, we showed that patients with strongly increased CBF in some regions (e.g., parietal lobes, temporal lobes, basal ganglia, DWM, and NAWM) had worse EDSS scores or higher sNfL than patients with mildly increased CBF, and that patients with high EDSS scores or sNfL had increased CBF compared to those with low EDSS scores or sNfL. Furthermore, strongly increased CBF group had more patients in relapsing phase. Thus, increased CBF suggests a high EDSS score, high sNfL, or acute attack.

Due to the operating complexity and requirements for advanced and expensive hardware or software, some quantitative imaging techniques have poor inter-scanner reproducibility and limited availability. ASL, which does not require intravenous administration of gadolinium-based contrast agents, is simple, noninvasive, and low-cost. As it enables the measurement of CBF with high reproducibility, it has good potential for clinical use. In summary, CBF is a potentially useful quantitative imaging marker associated with MS disease severity.

### Future directions

With pharmaceutical developments, there are increasing drugs available for MS. Therapeutic effects can be assessed by monitoring changes in clinical symptoms and neuroimaging lesions after administration of treatment. It is important that clinicians can predict future response to treatment early after disease onset and choose the most appropriate treatment. Since the present study indicates that CBF measurement could be used to monitor disease severity, it might also be applicable for assessment of treatment effect and aid clinicians in adjusting the treatment dose or treatment type. The identification of imaging markers with prognostic value for disability progression is also of considerable interest. CBF abnormalities may be observed before lesions appear on conventional MRI (23, 27), and our results indicate that relapsing probability might be higher in patients with mildly increased CBF in GM. Thus, CBF measurement has the potential to predict disease progression, prognosis, and treatment response. Due to the relative simplicity and reproducibility of the ASL technique, CBF measurement may have good prospects for clinical application and could be a useful complement to conventional MRI.

### Limitations

This study is subject to several limitations. First, as MS is relatively rare in China, the enrolled sample is not large enough. The sensitivity and effectiveness of CBF as a quantitative imaging marker requires further evaluation larger patient groups. Second, the value of CBF in predicting disease progression was not evaluated due to the lack of long-term follow-up. The longitudinal observation is needed to explore the predictive ability of CBF. Third, because of limited sample size some clinical parameters (e.g., different DMTs, cerebrospinal fluid NfL) were not included in the present study. In the future, we will recruit more MS patients and perform long-term follow-up to deepen our understanding of CBF.

## Conclusion

It is the first study to comprehensively depict the relationship between CBF changes and multiple clinical parameters in Eastern MS patients, enriching the data of CBF in this population. Increased CBF was observed in multiple brain regions, especially NAWM, parietal lobes, temporal lobes, and basal ganglia. Furthermore, there was a strong association between CBF and disease severity. These findings expand our understanding of CBF in MS and suggest that CBF has potential as a quantitative imaging marker associated with disease severity.

## Data availability statement

The original contributions presented in the study are included in the article/supplementary material. Further inquiries can be directed to the corresponding authors.

## Ethics statement

The studies involving human participants were reviewed and approved by Ethics Committee of Ruijin Hospital. The patients/participants provided their written informed consent to participate in this study.

## Author contributions

Conceptualization, SC, MZ and BL. Data curation, QZ, MZ. Formal analysis, QZ, TZ, HYM. Investigation, DS, LH, YG, YZ. Methodology, YL, XH, HPM. Project administration, SC and MZ. Writing – original draft, QZ, DS. Writing - review & editing, SC and MZ.

## Funding

This research was funded by Shanghai Shuguang Plan Project (18SG15), Shanghai outstanding young scholars Project, Shanghai talent development project (2019044), Clinical Research Plan of SHDC (SHDC 2020CR2027B) to SC, and Shanghai Municipal Key Clinical Specialty (shslczdzk03403).

## Conflict of interest

The authors declare that the research was conducted in the absence of any commercial or financial relationships that could be construed as a potential conflict of interest.

## Publisher’s note

All claims expressed in this article are solely those of the authors and do not necessarily represent those of their affiliated organizations, or those of the publisher, the editors and the reviewers. Any product that may be evaluated in this article, or claim that may be made by its manufacturer, is not guaranteed or endorsed by the publisher.

## Abbreviations

ARR, annualized relapse rate; ASL, arterial spin labeling; Basal_L, left basal ganglia; Basal_R, right basal ganglia; CBF, cerebral blood flow; Cereb_L, left cerebellum; Cereb_R, right cerebellum; DWM, damaged white matter; EDSS, Expanded Disability Status Scale; FA, flip angle; Fron_L, left frontal lobe; Fron_R, right frontal lobe; GM, grey matter; HC, healthy control; MRI, magnetic resonance imaging; MS, multiple sclerosis; NAWM, normal-appearing white matter; NAWM_L, left normal-appearing white matter; NAWM_R, right normal-appearing white matter; Occi_L, left occipital lobe; Occi_R, right occipital lobe; Pari_L, left parietal lobe; Pari_R, right parietal lobe; pASL, pulsed arterial spin labeling; sNfL, serum neurofilament light chain; TE, echo time; Temp_L, left temporal lobe; Temp_R, right temporal lobe; TI, inverse time; TR, repetition time; WM, white matter.
